# Posterior Cortical Atrophy Presenting with Superior Arcuate Field Defect

**DOI:** 10.1155/2015/796381

**Published:** 2015-08-31

**Authors:** Sue Ling Wan, Danuta M. Bukowska, Stephen Ford, Fred K. Chen

**Affiliations:** ^1^Centre for Ophthalmology and Visual Science (Incorporating Lions Eye Institute), The University of Western Australia, Nedlands, WA 6009, Australia; ^2^Department of Ophthalmology, Royal Perth Hospital, Perth, WA 6000, Australia; ^3^Department of Psychiatry, Sir Charles Gairdner Hospital, North Metropolitan Mental Health Service, Older Adult Program, Nedlands, WA 6009, Australia

## Abstract

An 80-year-old female with reading difficulty presented with progressive arcuate field defect despite low intraocular pressure. Over a 5-year period, the field defect evolved into an incongruous homonymous hemianopia and the repeated neuroimaging revealed progressive posterior cortical atrophy. Further neuropsychiatric assessment demonstrated symptoms and signs consistent with Benson's syndrome.

## 1. Introduction

Posterior cortical atrophy (PCA) is a neurodegenerative syndrome characterised by atrophy of the posterior cerebral cortex and disruption of higher visual functions. Here we describe a unique case of posterior cortical atrophy presenting with superior arcuate defect simulating early glaucoma.

## 2. Case Presentation

**Figure 1:**
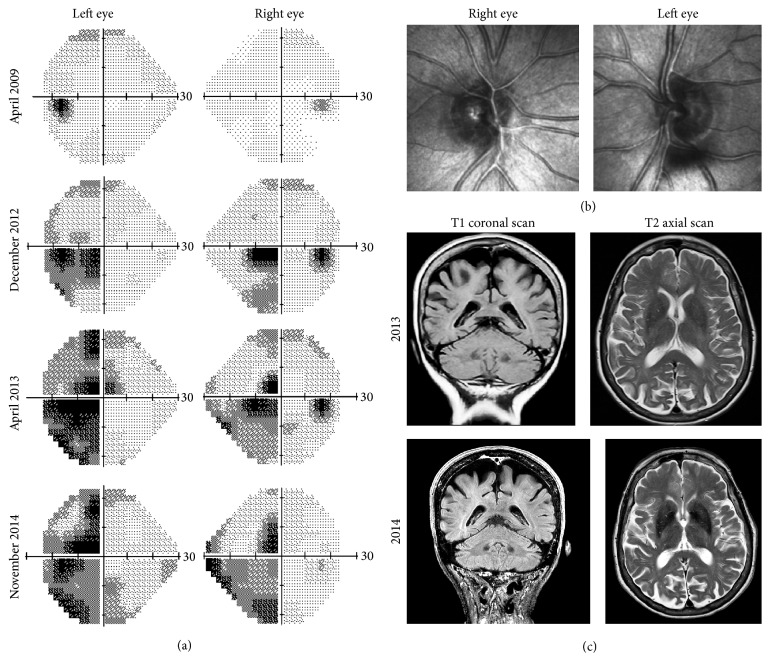
(a) Serial Humphrey visual fields from April 2009 to November 2014 showing progression of superior arcuate defect in the left eye to left-sided incongruous homonymous hemianopia. (b) Optic nerve head imaging in 2014 using the Heidelberg retinal tomography showing no significant disc cupping. (c) Coronal and axial magnetic resonance imaging (without contrast) in 2013 and 2014 showed progressive parietal and occipital cortical atrophy. Posterior cortical atrophy was suspected only in the 2014 scan.

**Figure 2:**
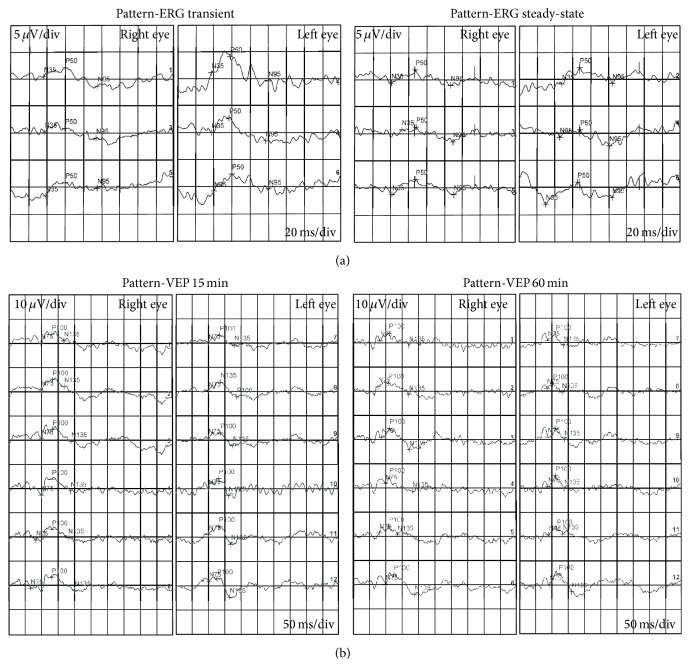
(a) Pattern electroretinography showing delayed P50 and N95 implicit times and only mild reduction in amplitude. (b) Pattern visual evoked potential showing significant reduction in amplitude.

A right-handed 80-year-old Caucasian female was referred for further evaluation of reading difficulty. She initially presented at age of 72 years with reduced vision and glare. She had no history of stroke or head injury. Following bilateral sequential phacoemulsification and insertion of intraocular lenses, she was diagnosed by a general ophthalmologist with low tension glaucoma in the left eye on the basis of unilateral superior arcuate field defect, temporal neuroretinal rim thinning, and a maximum intraocular pressure (IOP) of 17 mmHg at several clinic visits over an 18-month period (Figures 1(a)-1(b)). Timolol and then subsequently combined timolol-latanoprost eye drops were prescribed and IOP was reduced to around 12–15 mmHg. Despite good control over 4 years, the left visual field defect progressed and left sided homonymous hemianopia developed in the right visual field without significant change in the optic nerve heads (Figure 1(a)). Since she was also experiencing difficulty in reversing her car due to poor vision on the left-hand side, her antiglaucoma medication was ceased and ocular diagnosis reevaluated. Brain and orbital computer tomography (CT) scan showed no compressive lesion in the posterior visual pathway. Magnetic resonance imaging (MRI) of the brain and orbits (without contrast) demonstrated no mass or infiltrative lesion in the optic tracts or nerves that could explain the hemianopia. Although technically difficult, visual electrophysiology demonstrated delayed P50 and N95 implicit times with only mild reduction in amplitude on pattern electroretinography (PERG) whilst small and large check pattern visual evoked potentials (PVEP) were significantly reduced (Figure 2). At this point, further opinion was sought to exclude a retinal cause for her progressive visual field loss.

**Figure 3:**
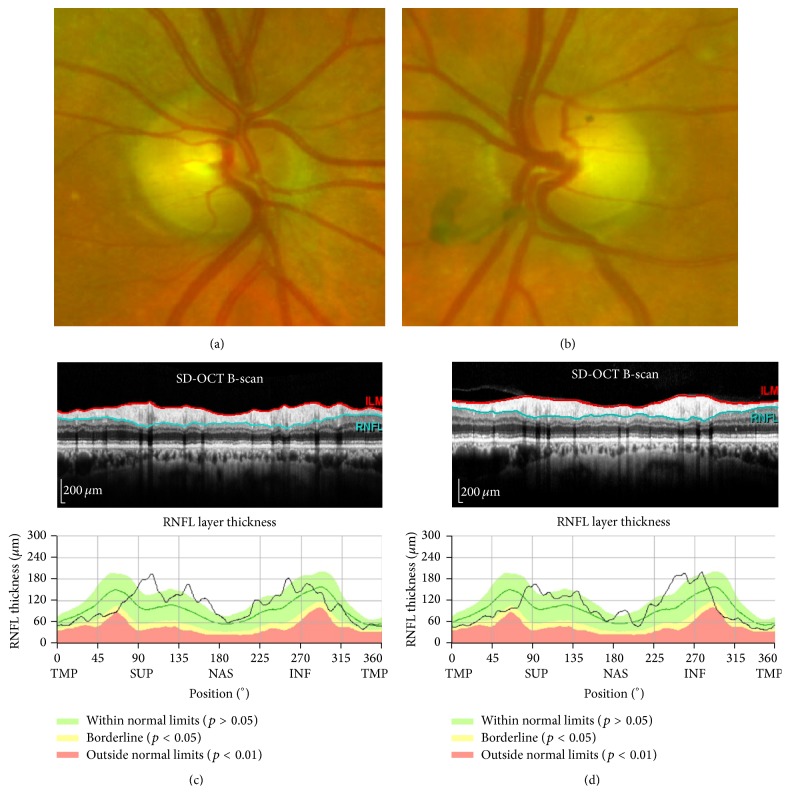
(a, b) Optic nerve head and (c, d) nerve fibre layer thickness imaging showing mild temporal pallor but no glaucomatous disc cupping and normal peripapillary nerve fibre thickness profile.

**Figure 4:**
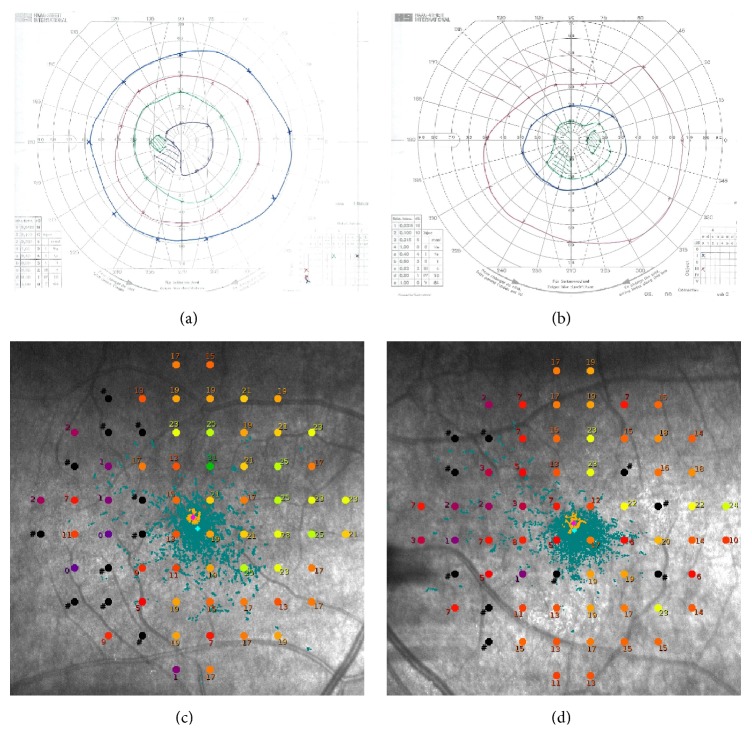
(a, b) Left and right Goldmann visual fields showing central incongruous homonymous hemianopia obeying the vertical midline. (c, d) Right and left MAIA microperimetry demonstrated diffuse reduced retinal sensitivity (abnormal sensitivity is < 25 dB) with nasal-temporal asymmetry.

Examination showed visual acuity of 6/9 in both eyes with IOPs of 10 and 11 mmHg in right and left eyes. Pupil reactions were slow and a definite afferent pupillary defect could not be elicited. Range of ocular motility was full but saccades were hypometric. She was unable to recognise any numbers on the Ishihara pseudoisochromatic charts despite being able to discriminate hues of colours by tracing out the numbers correctly from each eye separately using her dominant index finger. Slight temporal pallor of the discs was noted in both eyes and there was no glaucomatous disc cupping (Figures 3(a)-3(b)). Drusen were noted in the peripheral retina and retinal vessels and maculae were normal. Nerve fibre layer thickness on optical coherence tomography was within normal limits in each eye (Figures 3(c)-3(d)). Repeat Humphrey visual field testing consistently demonstrated an incomplete incongruous hemianopia (Figure 1(a)). Goldmann field showed hemianopic defect obeying the vertical midline whilst microperimetry (MAIA, CentreVue, Padova, Italy) demonstrated diffuse reduction in macular sensitivity with nasal-temporal asymmetry (Figure 4).

**Figure 5:**
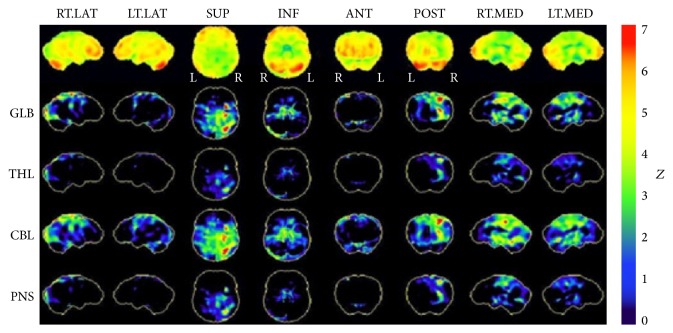
Single-photon-emission computed tomography (SPECT). Cerebral perfusion scan 99mTc HMPAO Z-score reconstructed images. Statistical mapping carried out using the NeuroSTAT programme. Demonstrating asymmetrically decreased activity involving the parietal, occipital, and, to a lesser extent, temporal lobe cortex, greater on the right than on the left. RT.LAT = right lateral; LT.LAT = left lateral; SUP = superior; INF = inferior; ANT = anterior; POST = posterior; RT.MED = right medial; LT.MED = left medial. GLB = global; THL = thalamus; CBL = cerebellum; PNS = pons.

The combination of number agnosia and statokinetic dissociation of the incongruous homonymous hemianopia redirected our attention to the occipital cortex. A repeat MRI scan confirmed parietal, occipital, and posterior temporal atrophy, more severe on the right side involving the right occipital lobe, suggesting a diagnosis of posterior cortical atrophy (PCA or Benson's syndrome). Comparison to previous MRI scan in 2013 showed deterioration in cortical atrophy (Figure 1(c)). Detailed psychogeriatric assessment revealed simultanagnosia, left visual neglect, constructional apraxia, number and image agnosia, altered colour perception, and mild memory and language deficits. She scored 78/100 on Addenbrooke's Cognitive Examination (version 3, normal > 82) with prominent deficits on the visuospatial (10/16) and the memory (23/26) subscales. On the Informant Questionnaire on Cognitive Decline in the Elderly (IQCODE, score = 3.75), her husband rated her as having deteriorated memory about family and friends and much worse for recent events. Functionally the main impairment was difficulty in judging distances and visual search. Cerebral perfusion scan demonstrated cognitive and perfusion deficits that were consistent with this diagnosis (Figure 5) and not consistent with typical Alzheimer's disease or Lewy body dementia.

## 3. Discussions

This case illustrates the evolution of PCA and the diagnostic dilemma faced by three ophthalmologists when PCA presents with nonspecific symptoms such as difficulty in reading and subtle visual field defect. The differential diagnosis for this patient's presentation includes low tension glaucoma, ischaemic optic neuropathy, and postchiasmal visual pathway diseases. The lack of neuroretinal rim thinning despite progressive field defect makes glaucoma unlikely. The abnormal Ishihara chart test result could be due to number agnosia as the patient was able to discriminate hues of colour by tracing out the numbers correctly using each eye separately. Further neuropsychiatric assessment showed that she was unable to name or distinguish colours placed adjacent to each other and this may be secondary to colour agnosia or cerebral achromatopsia. Although nonarteritic ischaemic optic neuropathy (NAION) can present with pallor of the optic nerve head, paracentral scotoma, and reduced colour vision, progression of incongruous homonymous hemianopia, hemifield visual neglect, and the development of simultanagnosia, constructional apraxia, and deficit of a variety of other cortical functions despite preservation of nerve fibre layer thickness suggests that PCA is a more likely diagnosis than NAION. The possibility of PCA was also supported by features on MRI and functional brain imaging. Although it took three neuroimaging sessions to confirm PCA, a comparison with prior scans showed that cortical atrophy was already present, but, to a lesser extent, in the occipital lobe. This highlights the importance of the treating physician in communicating with the radiologist to evaluate cortical atrophy as well as ruling out compressive lesion within the visual pathway when central visual loss is investigated by neuroimaging. However, diagnosing early stage PCA with structural neuroimaging by MRI is often difficult, and examination by a neurologist or psychologist is more important. Another unique aspect in our case study is the illustration of incongruous hemianopia using microperimetry. Furthermore, combining microperimetry with kinetic perimetry, we have also demonstrated statokinetic dissociation, a common feature of cortical visual loss.

PCA was first described in 1988 by Benson et al. [1]. Recent literature reviews have summarised the visual clinical features, neuroimaging, possible pathology, biology, and genetics of PCA [2–4]. Alzheimer's disease is the most common underlying cause of PCA in pathological studies although there is continuing debate on whether these are two separate conditions. PCA is a relatively rare condition and the visual manifestations are outlined in Table 1. Our case demonstrated that patients may not have obvious cortical atrophy on clinical imaging in the early stages. Greater awareness of the syndrome will improve identification of early symptoms of PCA and prevent unnecessary medical interventions.

## Figures and Tables

**Table 1:** Visual manifestations of posterior cortical atrophy [3].

Glare sensitivity	Impaired contrast sensitivity	Visual disorientation
Simultanagnosia	Alexia	Reverse-size phenomena

Optic ataxia	Visual crowding	Mirror sign

Optic apraxia	Prosopagnosia	Illusions of movement of static objects

Visual field deficits	Palinopsia	Akinetopsia

Complex visual hallucinations	Achromatopsia	180-degree upside down room tilt illusion

Impaired depth perception	Prolonged colour after images	
